# Elongation factor P restricts *Salmonella*’s growth by controlling translation of a Mg^2+^ transporter gene during infection

**DOI:** 10.1038/srep42098

**Published:** 2017-02-09

**Authors:** Eunna Choi, Soomin Choi, Daesil Nam, Shinae Park, Yoontak Han, Jung-Shin Lee, Eun-Jin Lee

**Affiliations:** 1 Department of Genetic Engineering and Graduate School of Biotechnology, College of Life Sciences, Kyung Hee University, Yongin 17104, South Korea; 2 Division of Microbiology, Department of Molecular Cell Biology, Samsung Biomedical Research Institute, Sungkyunkwan University School of Medicine, Suwon 16419, South Korea; 3 Department of Molecular Bioscience, College of Biomedical Science, Kangwon National University, Chuncheon 24341, South Korea

## Abstract

When a ribosome translates mRNA sequences, the ribosome often stalls at certain codons because it is hard to translate. Consecutive proline codons are such examples that induce ribosome stalling and elongation factor P (EF-P) is required for the stalled ribosome to continue translation at those consecutive proline codons. We found that EF-P is required for translation of the *mgtB* gene encoding a Mg^2+^ transporter in the *mgtCBR* virulence operon from the intracellular pathogen *Salmonella* *enterica* serovar Typhimurium. *Salmonella* lacking EF-P decreases MgtB protein levels in a manner dependent on consecutive proline codons located in the *mgtB* coding region despite increasing transcription of the *mgtCBR* operon via the *mgtP* open reading frame in the leader RNA, resulting in an altered ratio between MgtC and MgtB proteins within the operon. Substitution of the consecutive proline codons to alanine codons eliminates EF-P-mediated control of the *mgtB* gene during infection and thus contributes to *Salmonella*’s survival inside macrophages where *Salmonella* experiences low levels of EF-P. This suggests that this pathogen utilizes a strategy to coordinate expression of virulence genes by an evolutionarily conserved translation factor.

Mg^2+^ is involved in many important biological processes including coordinating nucleotides, stabilizing ribosome or membrane, and participating in many enzymatic reactions^1^. For pathogens, the ability to acquire Mg^2+^ or maintain Mg^2+^ homeostasis is critical to survive within a host and cause diseases. In the intracellular pathogen *Salmonella enterica* serovar Typhimurium, Mg^2+^ transport is tightly regulated by three distinct loci-*mgtA, mgtCBR*, and *corA* encoding MgtA, MgtB, and CorA Mg^2+^ transporters respectively^2^. A strain lacking all three Mg^2+^ transporters could not support growth unless medium was supplemented with at least 10 mM Mg^2+^ 3. Among them, MgtB orthologs occur in most limited phylogenetic distribution and appear to be associated with enteric bacteria replicating within warm-blooded host during infection cycle^2^,^4^,^5^. Moreover, the fact that the *mgtB* gene is located in the *Salmonella* pathogenicity islands 3 (SPI-3)^6^ supports the notion that MgtB might contribute to *Salmonella*’s pathogenicity. More recently, it was reported that the *mgtB* gene is required for virulence in *Yersinia pestis*^7^.

*Salmonella* MgtB is a Mg^2+^ -transporting P-type ATPase^8^ that typically induces a conformation change of the protein itself mediated by phosphorylation at a conserved aspartic acid residue during transport cycle^1^,^2^. MgtB is an inner membrane protein with 10 transmembrane domains and the conserved aspartic acid critical for the P-type ATPase protein family is located at the cytosolic face between 4^th^ and 5^th^ transmembrane segments^9^. In agreement with the notion that MgtB mainly mediates Mg^2+^ influx, transcription of the *mgtB* gene is induced in a low Mg^2+^ environment by the PhoP/PhoQ two-component system^10^, which is a major transcriptional regulator for *Salmonella* virulence^11^. In fact, the *mgtB* gene is a part of the *mgtCBR* operon and cotranscribed with two other genes, *mgtC* and *mgtR*, encoding the MgtC protein required for macrophage survival and mouse virulence and the MgtR short-peptide regulator for MgtC proteolysis respectively^12^,^13^,^14^. Because the *mgtB* gene is transcribed from a single promoter located upstream of the *mgtC* gene as a part of the *mgtCBR* polycistronic messages^15^, one might expect that *Salmonella* produces MgtC and MgtB proteins at similar levels. However, this is clearly not the case for the *mgtCBR* operon because the AmgR RNA is transcribed from the *mgtC-mgtB* intergenic region and alters MgtC and MgtB protein levels by preferentially degrading the *mgtC* part of the polycistronic messages^15^. Here we report that *Salmonella* achieves altered MgtC and MgtB protein levels in the *mgtCBR* operon by another strategy and the altered MgtC and MgtB protein levels contribute to *Salmonella* virulence.

During protein translation, a ribosome often slows down or stalls on transcripts depending on mRNA sequences. Consecutive proline codons are such sequences because the α-imino group of the proline codons constrains peptide bond formation between the two proline codons^16^ and thus induces ribosome stalling when it appears in a consecutive fashion^17^,^18^. Elongation factor P is specifically required for the stalled ribosome to resume translation^17^,^18^ by promoting peptide bond formation between peptidyl-prolyl-tRNA at the ribosomal P site and incoming prolyl-tRNA at the A site^19^. Therefore, a presence or absence of EF-P controls expression of genes harboring consecutive proline codons by limiting translation and the proteins with the consecutive proline codons are expected to be less abundant in a strain lacking EF-P. Alternatively or in addition, EF-P controls transcription of genes preceded by a leader RNA if the leader RNA harbors a short open reading frame with consecutive proline codons and the sequences associated with the short ORF have a potential to form two sets of stem-loop structures, one of which functions as a transcription attenuator. Although bacterial transcription and translation are usually coupled, the presence of the consecutive proline codons that induce ribosome stalling could uncouple transcription and translation. In this particular case, the presence or absence of EF-P determines whether or not the ribosome stalls at the consecutive proline codons of the ORF in the leader RNA, thereby coupling/uncoupling between transcription of the leader RNA and translation of the short ORF within the leader. By doing so, EF-P controls the formation of the transcription attenuator that prevents transcription elongation into the downstream genes. EF-P controls transcription elongation of the *mgtCBR* operon by such a transcription attenuation-like mechanism via the 296 nt-long leader RNA harboring *mgtP* with three consecutive proline codons (Fig. 1)^20^,^21^. *Salmonella* lacking EF-P induces ribosome stalling at the consecutive proline codons in *mgtP* and allows the formation of stem-loop E structure, which enhances *mgtCBR* transcription (Fig. 1)^21^. This makes physiological sense because *Salmonella* indeed decreases EF-P mRNA levels during infection^21^ and it explains why the *mgtC* gene is highly expressed among other PhoP-regulated genes inside macrophages^22^,^23^.

In this paper, we described another layer of regulation in the *mgtCBR* operon mediated by EF-P. *Salmonella* lacking EF-P decreases MgtB protein levels via two consecutive proline codons located in the *mgtB* coding region despite increasing transcription of the entire *mgtCBR* operon by the leader RNA. Removal of EF-P-mediated control in the MgtB protein levels promotes *Salmonella*’s pathogenicity, implying that the ability to transport Mg^2+^ must be compromised during the course of infection.

## Results

### Inactivation of *efp* derepresses transcription of the *mgtCBR* operon by the leader mRNA harboring *mgtP*

**Figure 1:**
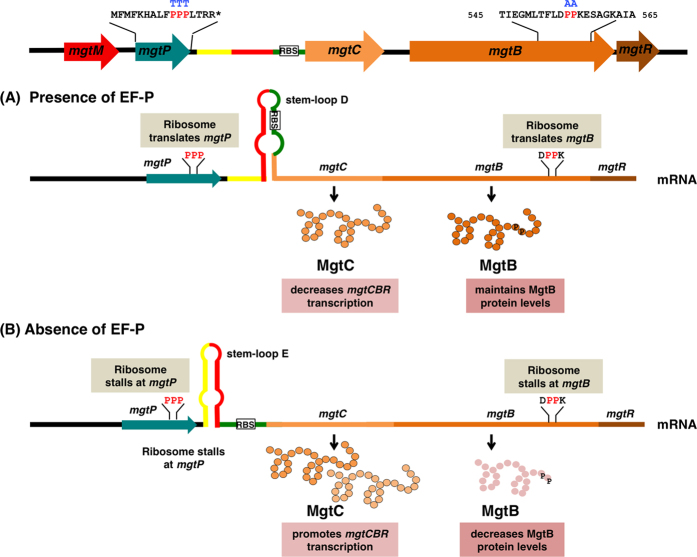
Regulation of the *mgtCBR* virulence operon by elongation factor P. EF-P controls expression of the *mgtCBR* operon in two different ways. On the one hand, EF-P controls transcription elongation of the *mgtCBR* operon by the leader RNA harboring the proline-rich short open reading frame *mgtP*. Three consecutive proline codons in *mgtP* induce ribosome stalling and require EF-P to continue translation. EF-P-mediated control in translating *mgtP* allows to from one of two alternative stem-loop structures (stem-loops D versus E) that control transcription elongation into the downstream region. On the other hand, EF-P controls translation of the *mgtB* part of the *mgtCBR* polycistronic messages by two consecutive proline codons located in the *mgtB* coding region. Therefore, when EF-P is absent or present in low levels (**B**), *Salmonella* promotes *mgtCBR* transcript levels by inducing ribosome stalling at the proline codons in *mgtP* and thus allowing the formation of stem-loop E structure. However, at the same time, *Salmonella* lacking EF-P decreases MgtB production by inducing ribosome stalling at the proline codons located in the *mgtB* coding region, thereby resulting in high MgtC: MgtB ratios compared to *Salmonella* producing EF-P (**A**). The sequences of the *mgtP* and *mgtB* variants used in this work are indicated above the Pro codons.

The *mgtCBR* leader RNA harbors *mgtP*, an 18 amino-acid long ORF with three consecutive proline codons (Fig. 1). Because *mgtP* is located adjacent to a sequence adopting stem-loop E structure (Fig. 1), the presence of the consecutive proline codons that induce ribosome stalling allows to form stem-loop E structure and promotes transcription into the downstream coding region. Therefore, *efp* inactivation was expected to induce transcription of the *mgtC* and *mgtB* genes in a manner dependent on consecutive proline codon at *mgtP.* Indeed, when grown in media containing 500 μM Mg^2+^ for 1 h to initiate transcription from the PhoP-dependent promoter, the *efp* mutant increased mRNA levels of both the *mgtC* and *mgtB* genes by ~100 fold (Fig. S1) and derepression in mRNA levels were not detected in a derivative strain with the *mgtP* proline codons substituted by threonine codons (Fig. S1).

### Loss of *efp* decreases MgtB protein levels in a PhoP/PhoQ-inducing condition

**Figure 2:**
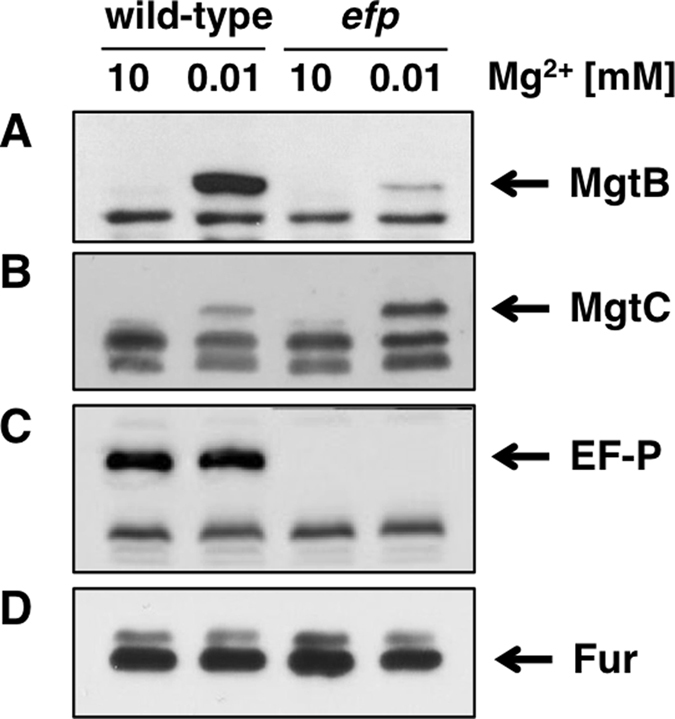
*Salmonella* lacking EF-P decreases MgtB protein levels despite increasing MgtC protein levels. Western blot analysis of crude extracts prepared from either wild-type (14028s) or the *efp* mutant *Salmonella* (DN337). Blots were probed with anti-MgtB (**A**), anti-MgtC (**B**), anti-EF-P (**C**), or anti-Fur (**D**) antibodies to detect MgtB, MgtC, EF-P, and Fur proteins respectively. Bacteria were grown for 5 h in N-minimal media containing 10 mM or 0.01 mM Mg^2+^ as described in Methods.

We assumed that *Salmonella* lacking EF-P would produce higher amounts of MgtB proteins based on the findings that *efp* inactivation increased transcription of the *mgtC* and *mgtB* genes (Fig. S1) and produced higher amounts of the MgtC proteins^21^ and that the *mgtC* and *mgtB* genes are transcribed from a single promoter located upstream of the *mgtC* gene^15^. To test this assumption, we measured the amounts of the MgtB proteins in low Mg^2+^ media to activate transcription of the *mgtCBR* operon by the PhoP/PhoQ two-component system^10^. Strikingly, MgtB proteins were barely detected in the *efp* mutant compared to those in the wild-type *Salmonella* (Fig. 2A). This is in contrast with the MgtC proteins that were produced in a higher amount in the *efp* mutant (Fig. 2B)^21^. Control experiments were carried out as follows: Both MgtB and MgtC proteins were not detected in a non-inducing media containing 10 mM Mg^2+^ (Fig. 2A and B), *efp* inactivation eliminates EF-P production (Fig. 2C), and Fur protein levels, which were used as loading control, were unaffected in all growth conditions (Fig. 2D).

### The consecutive proline codons at positions 555 and 556 are required for EF-P-mediated MgtB production

**Figure 3:**
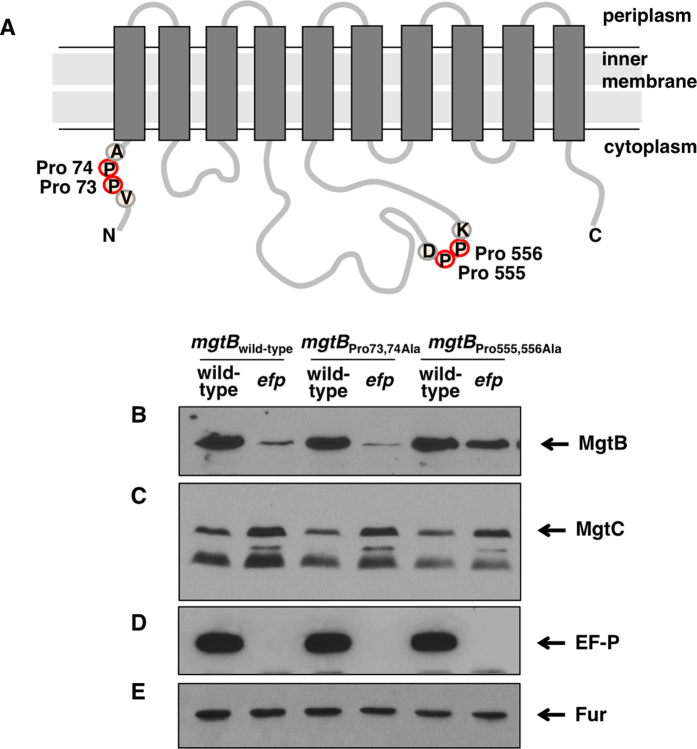
EF-P is required for MgtB production in a manner dependent on Pro^555^ and Pro^556^ codons. (**A**) Schematic diagram of the MgtB protein and location of proline codons used in this work. (**B–D**) Western blot analysis of crude extracts prepared from a strain with the wild-type *mgtB* gene (14028s), the *efp* mutant (DN337), an *mgtB* derivative with the Pro 73, 74 codons substituted by Ala codons (EN793), a mutant with both the Pro 73, 74 substitution and the *efp* insertion (EN794), an *mgtB* derivative with the Pro 555, 556 codons substituted by Ala codons (EN873), or a mutant with both the Pro 555, 556 substitution and the *efp* insertion (EN877). Blots were probed with anti-MgtB (**B**), anti-MgtC (**C**), anti-EF-P (**D**) or anti-Fur (**E**) antibodies to detect MgtB, MgtC, EF-P and Fur proteins respectively. Bacteria were grown for 5 h in N-minimal media containing 0.01 mM Mg^2+^ as described in Methods.

Given that EF-P’s role is limited to rescue stalled ribosome at consecutive proline codons^17^,^18^, low levels of MgtB in the *efp* mutant indicate that the *mgtB* gene might have consecutive proline codons responsible for EF-P-mediated control. We searched for the presence of consecutive proline codons in the *mgtB* coding region. Amino acid sequence analysis revealed that the *mgtB* gene has two pairs of consecutive proline codons in the coding region. One is located at positions 73 and 74 in the N-terminal cytoplasmic region of the MgtB protein and the other is located at positions 555 and 556 in the cytoplasmic loop region between 4^th^ and 5^th^ transmembrane domains (Fig. 3A). To determine which consecutive proline codons are required for EF-P-mediated MgtB expression, we created chromosomal mutant strains where respective two consecutive proline codons were replaced with alanine codons. Then, we compared MgtB protein levels of each mutant in either the wild-type and *efp* mutant background. When grown in low Mg^2+^ media, the Pro 555, 556 Ala substitution resisted to decrease MgtB levels despite *efp* inactivation, indicating that proline codons at positions 555 and 556 are critical for EF-P-mediated control of the *mgtB* gene (Fig. 3B). By contrast, a *Salmonella* mutant strain with the Pro 73, 74 Ala substitution behaved just like the isogenic strain with the wild-type *mgtB* gene (Fig. 3B). It is consistent with the previous notion that adjacent or neighboring sequences affect how strongly the ribosome stalls at those consecutive proline codons because Pro 555, 556 codons preceded by Asp 554 were strongly affected by EF-P while Pro 73, 74 codons preceded by Val 72 were not^24^,^25^,^26^. Control experiments proved that substitutions at consecutive proline codons in the *mgtB* gene did not affect the expression behavior of MgtC (Fig. 3C, EF-P (Fig. 3D, and Fur (Fig. 3E) protein levels.

### *Salmonella* with the *mgtB* Pro 555, 556 substitution carries a Mg^2+^ -transporting activity in a strain lacking other Mg^2+^ transporters

**Figure 4:**
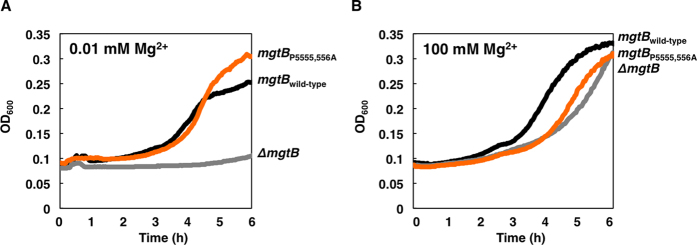
MgtB with the Pro 555, 556 substitution supports growth in low Mg^2+^ in a strain lacking other Mg^2+^ transporters. (**A,B**) Growth curves of strains with the wild-type *mgtB* gene (EL496), the *mgtB* derivative with the Pro 555, 556 codons substituted by Ala codons (EN943), or the *mgtB* insertion (EL498) in a genetic background where both the *corA* and *mgtA* genes are deleted. Bacteria were grown in N-minimal medium containing 0.01 mM (**A**) or 100 mM Mg^2+^ (**B**) at 37 °C for 6 h in a 96-well plate with orbital shaking and measured absorbance at OD_600_ every 2.5 min.

The *mgtB* gene encodes a Mg^2+^ transporting P-type ATPase with 10-transmenbrane segments and the Pro 555 and 556 residues are located in the large cytoplasmic loop that lies between transmembrane 4 and 5^9^. Because previous amino acid sequence analysis implicated that Asp 554 seems to be involved in ATP binding necessary for Mg^2+^ transport^8^, we wondered whether the Pro 555, 556 Ala substitution affects MgtB’s ability to transport Mg^2+^. To explore this, we created isogenic strains where two other Mg^2+^ transporter genes, *corA* and *mgtA*, were inactivated in either the wild-type *mgtB* or the *mgtB* derivative *Salmonella* with the Pro 555, 556 Ala substitution in its original chromosomal location. Given that growth of the *corA* and *mgtA* double knockout strain would be dependent on only functional MgtB, we tested MgtB’s activity to transport Mg^2+^ by determining ability to grow in low Mg^2+^ media. The *mgtB* derivative with the Pro 555, 556 Ala substitution grew well just like the isogenic strain with the wild-type *mgtB* gene in low Mg^2+^ media (Fig. 4A), demonstrating that MgtB with the Pro 555, 556 Ala substitution is functional for transporting Mg^2+^ to support growth in low Mg^2+^ media. By contrast, an introduction of *mgtB* deletion to the isogenic strain generated a triple knockout strain lacking all three Mg^2+^ transporters in *Salmonella* and *Salmonella* lacking all three Mg^2+^ transporters could not support growth in low Mg^2+^ media (Fig. 4A). As a control, 100 mM Mg^2+^ supplement in the liquid media restored growth of all strains (Fig. 4B).

### EF-P affects normal MgtB expression when *Salmonella* is inside macrophages

**Figure 5:**
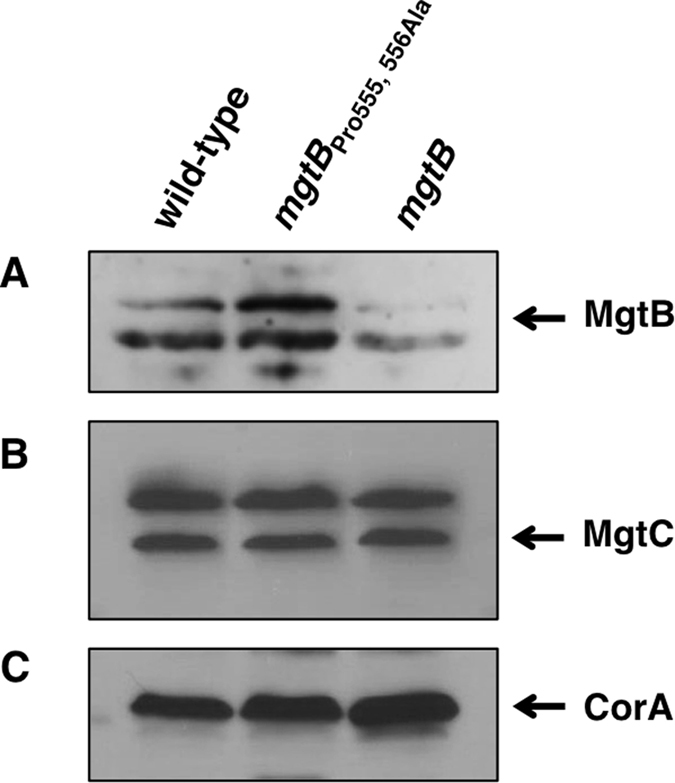
*Salmonella* with the *mgtB* Pro 555, 556 Ala substitution produces higher MgtB protein amounts inside macrophages. Western blot analysis of crude extracts prepared from wild-type (14028s), the *mgtB* derivative with the Pro 555, 556 codons substituted by Ala codons (EN873) or an *mgtB* deletion mutant (EN481) *Salmonella* inside J774 A.1 macrophage-like cells 21 h after infection. The amounts of MgtB (**A**) and MgtC (**B**) proteins were determined by anti-MgtB or anti-MgtC antibodies. Anti-CorA antibodies were used to detect CorA proteins (**C**) as loading controls.

The results described above indicate that EF-P controls translation of the *mgtB* gene via consecutive proline codons at positions 555 and 556. We wondered what the physiological relevance of EF-P-mediated control in the *mgtB* gene would be. Because the mRNA levels of EF-P decrease during the course of infection^21^, one might expect that *Salmonella* would decrease MgtB protein levels inside macrophages. And, if this is the case, the *mgtB* derivative with the Pro 555, 556 Ala substitution would not be subject to EF-P-mediated control and the levels of MgtB will remain high even inside host cells. To explore this possibility, we measured MgtB protein amounts in the wild-type or the *mgtB* derivative *Salmonella* inside J774 A.1 macrophages. As expected, the *mgtB* derivative strain with the Pro 555, 556 Ala substitution produced higher levels of MgtB proteins compared to those of the wild-type at 21 h after infection (Fig. 5A). Consistent with previous observations, EF-P appeared to control MgtB expression at the level of translation because the mRNA levels of the wild-type and *mgtB* derivative with the Pro 555, 556 substitution were similar inside macrophages (Fig. S2). Control experiments proved that: first, *mgtB* inactivation abolished MgtB production (Fig. 5A). Second, the levels of MgtC and CorA proteins were similar in the wild-type and *mgtB* derivative strains (Fig. 5B and C). Therefore, this suggests that, under normal circumstances, *Salmonella* decreases MgtB expression inside macrophages by limiting translation of *mgtB* mRNAs via low levels of EF-P.

### The *mgtB* Pro 555, 556 Ala mutant *Salmonella* displays a hypervirulent phenotype in intramacrophage survival and mouse virulence

**Figure 6:**
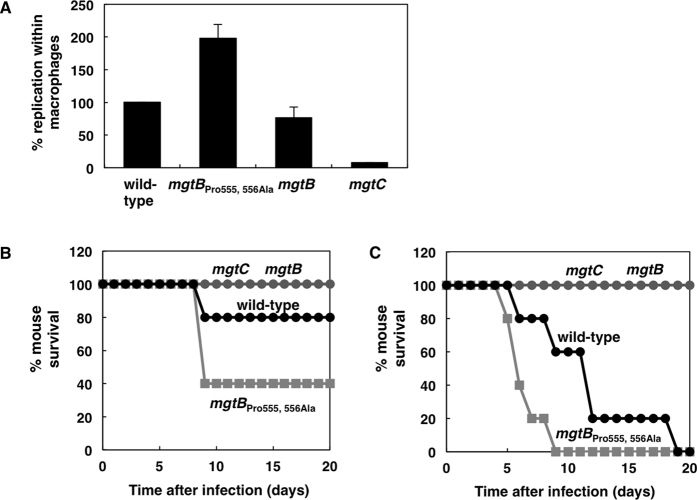
The *mgtB* Pro 555, 556 Ala substitution promotes *Salmonella*’s survival inside macrophages and virulence in mice. (**A**) Replication inside J774 A.1 macrophages of wild-type (14028s), the *mgtB* derivative with Pro codons replaced by Ala codons (EN873), the *mgtB* deletion mutant (EN481), or an *mgtC* deletion mutant (EL4) *Salmonella* at 21 h after infection. (**B,C**) Survival of C3H/HeN mice inoculated intraperitoneally with ~200 (**B**) or ~3000 (**C**) colony forming units of the *Salmonella* strains listed above.

If *Salmonella* decreases EF-P mRNA levels during infection, low levels of EF-P would induce ribosome stalling at three consecutive proline codons of *mgtP* located in the leader RNA and thus increase transcription of the *mgtCBR* operon. This explains in part why transcription of the *mgtC* gene is highly induced inside macrophages^20^,^22^. It also makes sense because the *mgtC* gene is required for survival inside macrophages and virulence in mice^6^,^14^. At the same time, however, low levels of EF-P would decrease translation of the *mgtB* part of the *mgtCBR* polycistronic messages via two consecutive proline codons located in the *mgtB* coding region (Fig. 3). If this is the case, it will create a situation where *Salmonella* decreases MgtB production while still increases MgtC production inside host cells. We wondered whether the decrease in MgtB levels via EF-P has a physiological impact on *Salmonella*’s ability to replicate within macrophages. To address this question, we measured survival inside macrophage-like J774A. 1 cell line using the *Salmonella mgtB* derivative with the Pro 555, 556 Ala substitution because it is not subjected to EF-P-mediated control and it produced higher amounts of MgtB protein inside macrophages (Fig. 5). The Pro 555, 556 Ala substitution increased *Salmonella*’s replication inside macrophages by nearly 200% relative to that of wild-type *Salmonella* (Fig. 6A), suggesting that enhanced production of MgtB proteins promotes *Salmonella*’s survival inside a host, presumably by increasing the ability to uptake Mg^2+^ from the host. By contrast, the *mgtB* deletion mutant did not have a significant defect in intramacrophage survival (Fig. 6A). As a control, the *mgtC* mutant showed a remarkable defect in intramacrophage survival (Fig. 6A) as previously described^6^,^27^. Similar to those from intramacrophage survival, when we injected *Salmonella* strains listed above into mice intraperitoneally, the *mgtB* Pro 555, 556 Ala substitution renders *Salmonella* hypervirulent (Fig. 6B and C). These results showed that EF-P-mediated control of MgtB protein levels is critical for *Salmonella* virulence.

## Discussion

Here we established that *Salmonella* decreases MgtB protein levels by lowering EF-P levels during infection. Such a decrease in MgtB levels mediated by EF-P is dependent on the consecutive proline codons located in the *mgtB* coding region (Fig. 3) and the decrease in MgtB protein levels is required for *Salmonella*’s ability to survive within macrophages and mouse virulence (Fig. 6). It is interesting to discover the decrease in MgtB levels inside host cells because low levels of EF-P during *Salmonella* infection actually *induce* transcription of the entire *mgtCBR* operon via consecutive proline codons at *mgtP* located in the *mgtCBR* leader RNA (Fig. 1)^21^. Then, one can easily imagine that *Salmonella* increases MgtC protein production but decreases MgtB’s during the course of infection, establishing high MgtC: MgtB protein ratios inside host cells. *Salmonella* seems to limit proliferation inside macrophages by decreasing the production of the Mg^2+^-importing MgtB transporter because substitution of the consecutive proline codons in the *mgtB* coding region that prevents downregulation of MgtB protein levels promotes *Salmonella*’s pathogenicity both in terms of macrophage survival and mouse virulence (Fig. 6). We wondered why *Salmonella* achieves differential protein levels between MgtC and MgtB within the same operon. We speculated that it might be due to homeostasis between ATP and Mg^2+^ levels during infection because the MgtC protein inhibits proton translocation of F_1_F_o_ ATP synthase within an acidified phagosome to promote *Salmonella*’s pathogenicity, resulting in a decrease in ATP production^27^. Given that Mg^2+^ is required to coordinate ATP for neutralizing its negative charges, the decrease in ATP production via enhanced MgtC protein levels could be compromised by the decrease in the ability to transport Mg^2+^ ion via the decrease in MgtB protein levels in the host environment.

EF-P-mediated differential regulation that takes place in the *mgtCBR* operon has several properties in common with those achieved by the AmgR RNA in the same operon. First, differential regulation within the operon is mediated by the same proteins. We described here that EF-P controls transcription elongation of the *mgtCBR* operon and, at the same time, controls translation of the *mgtB* part of the mRNA message. Similarly, PhoP controls transcription of the *mgtCBR* full-length messages as well as the AmgR antisense transcript initiated from *mgtC*-*mgtB* intergenic region toward the *mgtC* gene^15^. Second, mutations that prevent differential regulation promote *Salmonella*’s pathogenicity. The substitution that eliminates EF-P-mediated control in the *mgtB* gene increases MgtB protein levels, resulting in elevated proliferation inside macrophages and hypervirulence in mice. Likewise, the substitution that removes the promoter region of the *amgR* accumulates preferentially MgtC proteins and, to a lesser extent, MgtB proteins, leading to a hypervirulence phenotype in mice^15^. However, they clearly differ from each other in terms of how they achieve differential regulation within the same operon because EF-P does its task by controlling ribosome stalling on the mRNA messages whereas AmgR by degrading mRNA in an RNase E-mediated antisense mechanism. Moreover, PhoP binds the promoter of the *mgtCBR* operon with a higher affinity than that of the *amgR*^15^, gradually establishing different levels between MgtC and MgtB proteins inside host cells. This is in contrast with the fact that low levels of EF-P enable to maintain a steady-state MgtC: MgtB ratio during infection.

For *Salmonella* Typhimurium, ability to survive and replicate within a phagosome inside host macrophages is critical to cause diseases^28^. EF-P seems to be required for *Salmonella* virulence based on following: Inactivation of two genes specifying proteins required for EF-P’s activity^17^,^18^,^29^ is highly attenuated for mouse virulence^29^. And also a previous proteomic approach identified that many virulence genes were upregulated in a strain lacking functional EF-P^29^. Because EF-P is involved in translation of many genes with a variety of physiological functions^17^,^18^,^30^, it has been an intriguing question to figure out which genes are responsible for the virulence phenotype of strains lacking functional EF-P. Given that EF-P’s role in virulence could be limited to genes with consecutive proline codons in the coding region or in the short ORF located in the preceding leader RNA, the simplest scenario is that a particular virulence gene(s) controlled by EF-P represents EF-P’s virulence phenotype. In other words, EF-P controls translation of the particular virulence gene with consecutive proline codons and thus the strain lacking a functional EF-P would decrease the abundance of the corresponding proteins, in turn, resulting in an avirulent phenotype. In this case, one can expect that a deletion mutant of the particular gene would behave similarly to the mutants of genes encoding EF-P itself or EF-P modifying enzymes. Moreover, substitution of consecutive proline codons in the particular gene that eliminates EF-P-mediated control is expected to suppress the defect in the virulence phenotype of the strain lacking functional EF-P. However, the findings how EF-P controls expression of the *mgtCBR* operon and contributes to *Salmonella*’s pathogenicity imply that the virulence phenotype of the strain lacking functional EF-P is not so simple as we hypothesized. Low levels of EF-P during infection promote *Salmonella* pathogenicity by inducing production of the MgtC virulence protein via consecutive proline codons at *mgtP* in the leader RNA^21^ but, at the same time, limit the pathogen’s proliferation inside host by decreasing production of the MgtB Mg^2+^ transporter via another consecutive proline codons in the *mgtB* coding region. Substitution of proline codons in *mgtP* attenuates *Salmonella* virulence^31^ whereas substitution of proline codons in the *mgtB* coding region renders *Salmonella* hypervirulent (Fig. 6). Therefore, the virulence phenotype displayed by the strain lacking functional EF-P must be a reflection of the summation between two opposing effects caused by MgtC and MgtB proteins. And there are possibly more responsible genes out there.

## Methods

### Bacterial strains, plasmids, oligodeoxynucleotides and growth conditions

Bacterial strains and plasmids used in this study are listed in Table S1. All *Salmonella enterica* serovar Typhimurium strains are derived from the wild-type strain 14028s^32^ and were constructed by phage P22-mediated transductions as described^33^. All DNA oligonucleotides are listed in Table S2. Bacteria were grown at 37 °C in Luria-Bertani broth (LB), N-minimal media (pH 7.7)^8^ supplemented with 0.1% casamino acids, 38 mM glycerol and the indicated concentrations of MgCl_2_. *Escherichia coli* DH5α was used as the host for preparing plasmid DNA. Ampicillin was used at 50 μg ml^−1^, chloramphenicol at 25 μg ml^−1^, kanamycin at 50 μg ml^−1^, or tetracycline at 10 μg ml^−1^.

### Effect of *efp* inactivation on gene expression

Gene expression upon *efp* inactivation was measured as described previously^21^. Briefly, bacteria were grown overnight in N-minimal medium containing 10 mM Mg^2+^. 1/100 dilution of the overnight culture was used to inoculate 20 ml of the same medium and grown for 3 h. Cells were then washed and transferred to 20 ml of N-minimal medium containing 500 μM Mg^2+^ and grown for 1 h. Bacteria were stabilized using RNAprotect Bacteria Reagent (Qiagen) and RNA was isolated for further analysis.

### Quantitative real time-polymerase chain reaction (RT-PCR)

Total RNA was isolated using RNeasy Kit (Qiagen) according to the manufacturer’s instructions. The purified RNA was quantified using a Nanodrop machine (NanoDrop Technologies). cDNA was synthesized using PrimeScript^TM^ RT reagent Kit (TaKaRa). The mRNA levels of the *mgtC, mgtB, efp,* and *rrsH* genes were measured by quantification of cDNA using SYBR Green PCR Master Mix (TOYOBO) and appropriate primers (*mgtC*: 7530/7531, *mgtB*:7763/7764 and *efp*: qrt-efp-F/qrt-efp-R) and monitored using a 7300 Real Time PCR system (Applied Biosystems, Foster City). The mRNA levels of each target genes were calculated using a standard curve of 14028s genomic DNA with known concentration and data were normalized to the levels of 16S ribosomal RNA amplified with primers 6970 and 6971.

### Western Blot Analysis

Cells were grown for 5 h in 35 ml of N-minimal medium containing 10 μM Mg^2+^. Cells were normalized by measuring optical density at 600 nm (OD_600_). Crude extracts were prepared in PBS (Phosphate-buffered saline) buffer by sonication and analyzed as described^15^. The data are representative of two independent experiments, which gave similar results.

### Measuring growth of strains lacking Mg^2+^ transporters

*Salmonella* lacking all three Mg^2+^ transporters requires 100 mM Mg^2+^ in the medium to support growth^3^. To address whether proline to alanine substitution of the *mgtB* gene could support growth in a *Salmonella* strain lacking other two Mg^2+^ transporters, MgtA and CorA^34^, bacteria were grown in N-minimal medium containing 0.01 mM (Mg^2+^ limiting) or 100 mM Mg^2+^ (Mg^2+^ in excess). Growth was determined at 37 °C for 6 h in a 96-well plate with orbital shaking and absorbance measured at OD_600_ every 2.5 min.

### Examining survival inside macrophages

Intramacrophage survival assays were conducted with the macrophage-like cell line J774 A.1 as described^6^.

### Examining gene expression inside macrophages

Gene expression inside macrophages was measured as described previously^23^.

### Mouse virulence assays

Six- to eight-week-old female C3H/HeN mice were inoculated intraperitoneally with ~10^2^ or ~10^3^ colony-forming units. Mouse survival was followed for 21 days. Virulence assays were conducted twice with similar outcomes, and data correspond to groups of five mice. All procedures were performed according to approved protocols by the Institutional Animal Care and Use Committee from Kangwon National University.

### Construction of chromosomal mutant strains with the *mgtB* proline codons substituted by alanine codons

To generate strains with chromosomal mutations in the *mgtB* coding region, we used the fusaric acid-based counterselection method as described previously^15^. First, we introduced Tet^R^ cassettes in two different regions of the *mgtB* gene as follows: we generated PCR products harboring the *tetRA* genes using as template chromosomal DNA from strain MS7953s and primers KHU336/KHU337 (for 73^rd^ and 74^th^ proline codons) and KH472/KH473 (for 555^th^ and 556^th^ proline codons). The product was purified using a QIAquick PCR purification kit (QIAGEN) and used to electroporate *Salmonella* 14028s containing plasmid pKD46^35^. The resulting *mgtB* NT::*tetRA* (EN786) and *mgtB* 900 nt::*tetRA* (EN821) strains containing plasmid pKD46 were kept at 30 °C. Then, we replaced the *tetRA* cassettes by preparing DNA fragments carrying proline to alanine codons substitutions in *mgtB* at positions 73 and 74 or 555 and 556 were prepared by a two-step PCR process. For the first PCR, we used two sets of primer pairs 7554/KH379 and KH378/12605R (for 73^rd^ and 74^th^ proline codons) and KH476/KH475 and KH474/KH477 (for 555^th^ and 556^th^ proline codons), and 14028s genomic DNA as template. For the second PCR, we mixed the two PCR products from the first PCR as templates and amplified DNA fragments using primers 7554/12605R (for 73^rd^ and 74^th^ proline codons) and KH476/KH477 (for 555^th^ and 556^th^ proline codons). The resulting PCR products were purified and integrated into the EN786 and EN821 chromosome and selected against tetracycline resistance with media containing fusaric acid to generate EN793 (*mgtB*^Pro 73,74 Ala^) and EN873 (*mgtB*^Pro 555,556 Ala^), tetracycline-sensitive, ampicillin-sensitive (Tet^S^ Amp^S^) chromosomal mutants, respectively. The presence of the expected nucleotide substitutions was verified by DNA sequencing. A P22 phage lysate grown in strain DN337 was used to transduce strains EN793, and EN873 *Salmonella* selecting for chloramphenicol resistance to generate EN794 (*mgtB*^Pro 73,74 Ala^, *efp*::Cm^R^), and EN877 (*mgtB*^Pro 555,556 Ala^, *efp*::Cm^R^), respectively.

### Construction of strains with chromosomal deletions of the *mgtB* or *corA* genes

*Salmonella* strains deleted for the *mgtB* or *corA* genes were generated by the one-step gene inactivation method^35^. A Km^R^ cassette for the *mgtB* gene and a Cm^R^ cassette for the *corA* gene were PCR amplified from plasmid pKD4 or pKD3 using primers DE-*mgtB*-F/DE-*mgtB*-R (for *mgtB*), and del-corA-F/del-corA-R (for *corA*) and the resulting PCR products were integrated into the 14028s chromosome to generate EN480 (*mgtB*::Km^R^), and YS166 (*corA*::Cm^R^), respectively. The *mgtB* strain (EN481) was generated by removing the Km^R^ cassette from EN480 using plasmid pCP20 as described^35^. A P22 phage lysate grown in strain YS166 was used to transduce EG9521 *Salmonella* selecting for chloramphenicol resistance to generate EL496 (*corA::*Cm^R^, *mgtA*::MudJ). A P22 phage lysate grown in strain EL496 was used to transduce strains EN481, and EN873 *Salmonella* selecting for chloramphenicol and kanamycin resistance to generate EL498 (*corA::*Cm^R^, *mgtA*::Mu*d*J, Δ*mgtB*), and EN943 (*corA::*Cm^R^, *mgtA*::Mu*d*J, *mgtB*^Pro 555,556 Ala^), respectively.

## Additional Information

**How to cite this article:** Choi, E. *et al*. Elongation factor P restricts *Salmonella*’s growth by controlling translation of a Mg^2+^ transporter gene during infection. *Sci. Rep.* **7**, 42098; doi: 10.1038/srep42098 (2017).

**Publisher's note:** Springer Nature remains neutral with regard to jurisdictional claims in published maps and institutional affiliations.

## Supplementary Material

Supplementary Information
