# Supplementary material for: Elongation factor P restricts Salmonella’s growth by controlling translation of a Mg2+ transporter gene during infection
Source: Sci Rep. 2017 Feb 9;7:42098. doi: 10.1038/srep42098 (PMC5299641; doi:10.1038/srep42098)
Supplement: Supplementary Information [file srep42098-s1.pdf]

Supplementary information

Elongation factor P restricts *Salmonella*'s growth by controlling translation of a Mg^{2+} transporter gene during infection

Eunna Choi[a,b], Soomin Choi[a,b], Daesil Nam[c], Shinae Park[d], Yoontak Han[a], Jung-Shin Lee[d] and Eun-Jin Lee[a]*

Table S1. Bacterial strains and plasmids used in this study

Strain or plasmid	Description	Reference or source
***S. enterica* serovar Typhimurium**		
14028s	wild-type	[1]
EG9521	*mgtA9521*::Mu*d*J	[2]
MS7953s	*phoP7953*::Tn10	[3]
EL4	*mgtC*	[4]
EL611	*mgtP* $^{\mathrm{Pro}\rightarrow\mathrm{Thr}}$	[5]
EN480	*mgtB*::Km^{R}	This study
EN481	*mgtB*	This study
YS166	*corA*::Cm^{R}	This study
DN337	*efp*::Cm^{R}	[6]
DN353	*mgtP* $^{\mathrm{Pro}\rightarrow\mathrm{Thr}}$, *efp*::Cm^{R}	[6]
EN786	*mgtB* NT::*tetRA*	This study
EN821	*mgtB* 900 nt::*tetRA*	This study
EN793	*mgtB* $^{\mathrm{Pro\ 73,74\ Ala}}$	This study
EN794	*mgtB* $^{\mathrm{Pro\ 73,74\ Ala}}$, *efp*::Cm^{R}	This study
EN873	*mgtB* $^{\mathrm{Pro\ 555,556\ Ala}}$	This study
EN877	*mgtB* $^{\mathrm{Pro\ 555,556\ Ala}}$, *efp*::Cm^{R}	This study
EL496	*corA::*Cm^{R}, *mgtA*::Mu*d*J	This study
EL498	*corA::*Cm^{R}, *mgtA*::Mu*d*J, Δ*mgtB*	This study
EN943	*corA::*Cm^{R}, *mgtA*::Mu*d*J, *mgtB* $^{\mathrm{Pro\ 555,556\ Ala}}$	This study
**plasmids**		
pKD3	repR$_{6K\gamma}$ Ap^{R} FRT Cm^{R} FRT	[7]
pKD4	repR$_{6K\gamma}$ Ap^{R} FRT Km^{R} FRT	[7]
pKD46	rep$_{\mathrm{pSC101}}{}^{ts}$ Ap^{R} P$_{araBAD}$ γ β exo	[7]
pCP20	rep$_{\mathrm{pSC101}}{}^{ts}$ Ap^{R} Cm^{R} *cI857* λP$_{R}$*flp*	[7]

Table S2. Oligonucleotides used in this study

Primers	Sequences (5′ to 3′)
KHU336	TCGTCAGTGGTTACTGAATATCGTAAAAGAGGCCGCGATCTT AAGACCCACTTTCACATTTAAG
KHU337	CCCACGACGCAACACCGTGGCGGTGGTGCGAACCATCTTTCT AAGCACTTGTCTCCTGTTTAC
KH472	ATGATTGTCAGTTCCAACCTGGCAAAAGGCGCGATTGCGATT AAGACCCACTTTCACATTTAAG
KH473	TTTCTGTAGCGCTTGCAAAATACGCGTTTTTTGTAACGGCCTA AGCACTTGTCTCCTGTTTAC
KH476	CGCTTTATGCTGATCATGGTGCC
KH475	TTTCTTTGGCGGCATCGAGAAAAGT
KH474	ACTTTTCTCGATGCCGCCAAAGAAA
KH477	AATAATATCTGATGACTCTTTAG
7554	CAATATCCGCTGAGGGAGAA
12605R	TCGCCTGCGGCCAGAAAAAC
KH378	TGAGCAAGTTGCCGCCGCGCTGATTC
KH379	GAATCAGCGCGGCGGCAACTTGCTCA
DE-*mgtB*-F	ATATGCAGGAAACACTACACCTTAATTTTGGGGATTCATCTGT AGGCTGGAGCTGCTTCG

DE-*mgtB*-R	TATCGGGTGAGCGATTCATCTGGGCGATCCTCAAACATTACAT ATGAATATCCTCCTTAG
del-*corA*-F	CTGAACTGTCCGATATTTTTACGCATTGGGAGTCCCGGTCGTG TAGGCTGGAGCTGCTTC
del-*corA*-R	GGTGGTTCAGCCGCAGCTGAATCACCCTGGCCTTAATGTCATA TGAATATCCTCCTTAGT
7530	CAGCCCGCGCACATTC
7531	TTGTCTCTGGGATTGGCTTTCT
7763	TCAGAAAATGATAAGCAGCATAAAAAA
7764	CCCTGACGATGGCTGTTCA
qrt-efp-F	CGGTGAGTTCTGGCATTTCA
qrt-efp-R	CACAGGGTCACGATGCATTC
6970	CCAGCAGCCGCGGTAAT
6971	TTTACGCCCAGTAATTCCGATT

Supplementary references

1 Fields, P. I., Swanson, R. V., Haidaris, C. G. & Heffron, F. Mutants of *Salmonella typhimurium* that cannot survive within the macrophage are avirulent. *Proc Natl Acad Sci U S A* **83**, 5189-5193 (1986).

2 Soncini, F. C., Garcia Vescovi, E., Solomon, F. & Groisman, E. A. Molecular basis of the magnesium deprivation response in *Salmonella typhimurium*: identification of PhoP-regulated genes. *J Bacteriol* **178**, 5092-5099 (1996).

3 Fields, P. I., Groisman, E. A. & Heffron, F. A *Salmonella* locus that controls

resistance to microbicidal proteins from phagocytic cells. *Science* **243**, 1059-1062 (1989).

4 Lee, E. J., Pontes, M. H. & Groisman, E. A. A Bacterial Virulence Protein Promotes Pathogenicity by Inhibiting the Bacterium's Own F1Fo ATP Synthase. *Cell* **154**, 146-156, doi:10.1016/j.cell.2013.06.004 (2013).

5 Lee, E. J., Choi, J. & Groisman, E. A. Control of a Salmonella virulence operon by proline-charged tRNAPro. *Proc Natl Acad Sci U S A* **111**, 3140-3145, doi:10.1073/pnas.1316209111 (2014).

6 Nam, D., Choi, E., Shin, D. & Lee, E. J. tRNAPro -mediated downregulation of elongation factor P is required for mgtCBR expression during Salmonella infection. *Mol Microbiol*, doi:10.1111/mmi.13454 (2016).

7 Datsenko, K. A. & Wanner, B. L. One-step inactivation of chromosomal genes in *Escherichia coli* K-12 using PCR products. *Proc Natl Acad Sci U S A* **97**, 6640-6645 (2000).

Supplementary figure legends

Fig. S1. *Salmonella* lacking EF-P promotes transcription of the *mgtC* and *mgtB* genes in a manner dependent on the *mgtP* proline codons located in the leader RNA.

(*A*-*C*) Relative mRNA levels of the coding regions of the *mgtB* (*A*), *mgtC* (*B*), and *efp* (*C*) genes produced in a strain with the wild-type *mgtCBR* leader (14028s), an *efp* mutant

(DN337), a derivative with the three *mgtP* Pro codons substituted by Thr codons (EL611), or a mutant with both the *mgtP* substitution and the *efp* insertion (DN353). Bacteria were grown in N-minimal media with 10 mM Mg^{2+} for 3 h, and then grown for 1 h in the same media containing 500 μM Mg^{2+}. Expression levels of target genes were normalized to that of 16S ribosomal RNA *rrsH* gene. Shown are the mean and SD from two independent experiments.

Fig. S2. The mRNA levels of *Salmonella* with the *mgtB* Pro 555, 556 Ala substitution were similar to those of wild-type inside macrophages.

(*A*-*B*) Relative mRNA levels of the *mgtB* and *mgtC* coding regions produced by wild-type *Salmonella* (14028s), the *mgtB* derivative with the Pro codons replaced by Ala codons (EN873), or the *mgtB* mutant (EN481), inside J774 A.1 macrophages at the indicated times after infection.

Figure S1

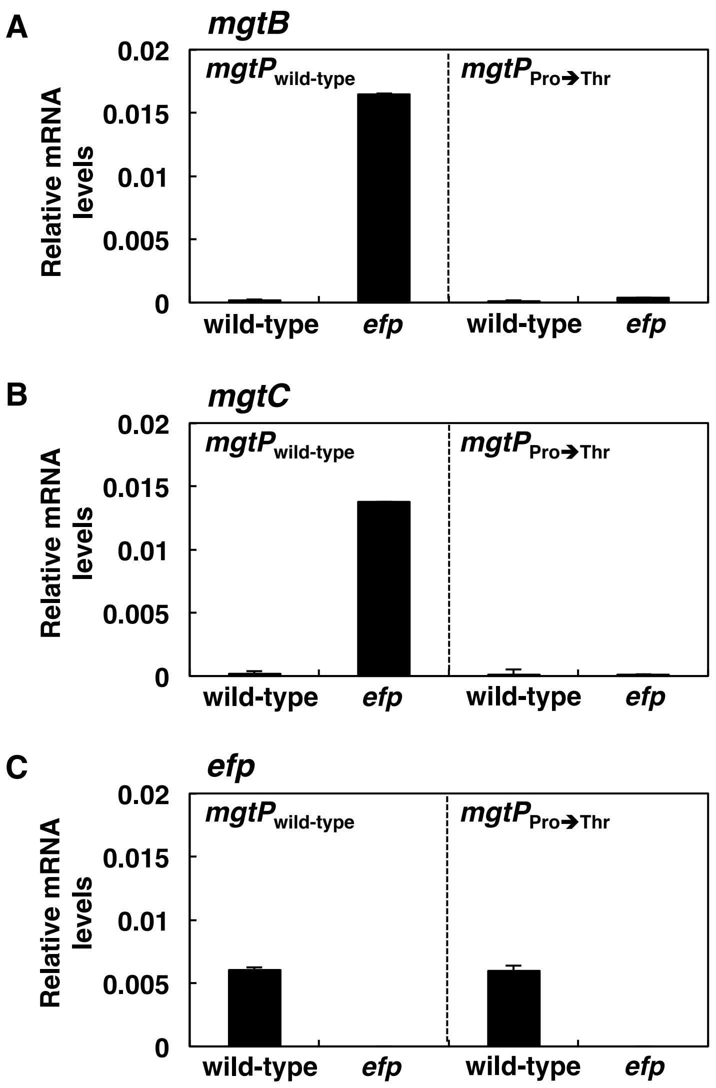
A
mgtB
B
mgtC
C
efp
Relative mRNA levels
0.02
0.015
0.01
0.005
0
mgtP wild-type
mgtP Pro→Thr
wild-type
efp
wild-type
efp

Figure S2

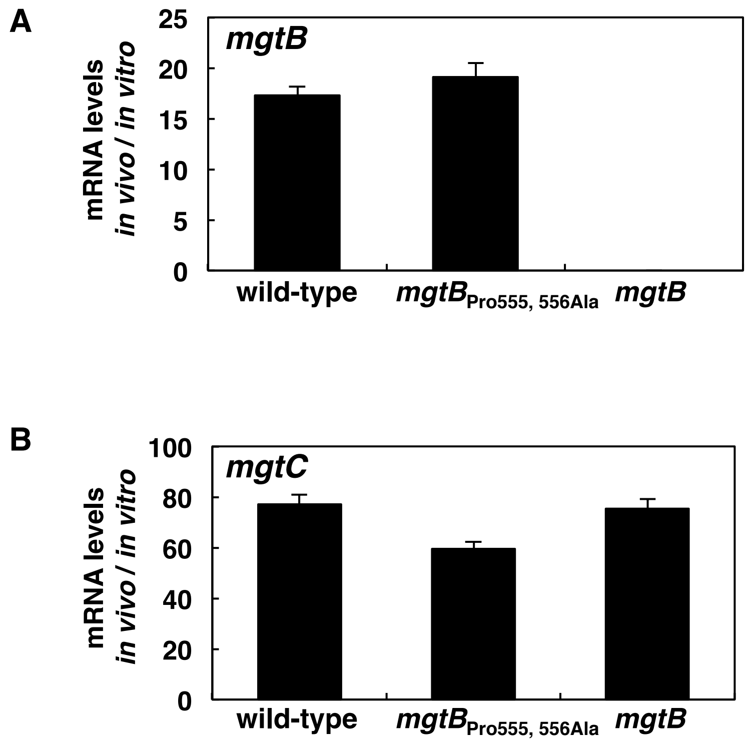
A
mgtB
mRNA levels
in vivo / in vitro
25
20
15
10
5
0
wild-type
mgtBPro555, 556Ala
mgtB
B
mgtC
mRNA levels
in vivo / in vitro
100
80
60
40
20
0
wild-type
mgtBPro555, 556Ala
mgtB
